# Survival of Virus Particles in Water Droplets: Hydrophobic Forces and Landauer’s Principle

**DOI:** 10.3390/e23020181

**Published:** 2021-01-30

**Authors:** Edward Bormashenko, Alexander A. Fedorets, Leonid A. Dombrovsky, Michael Nosonovsky

**Affiliations:** 1 Department of Chemical Engineering, Biotechnology and Materials, Engineering Science Faculty, Ariel University, Ariel 40700, Israel; edward@ariel.ac.il; 2 X-BIO Institute, University of Tyumen, 6 Volodarskogo St, 625003 Tyumen, Russia; fedorets_alex@mail.ru (A.A.F.); ldombr@yandex.ru (L.A.D.); 3 Joint Institute for High Temperatures, 17A Krasnokazarmennaya St, 111116 Moscow, Russia; 4 Department of Mechanical Engineering, University of Wisconsin–Milwaukee, 3200 North Cramer St, Milwaukee, WI 53211, USA

**Keywords:** viruses, bioinformatics, information, droplet cluster, Landauer’s principle

## Abstract

Many small biological objects, such as viruses, survive in a water environment and cannot remain active in dry air without condensation of water vapor. From a physical point of view, these objects belong to the mesoscale, where small thermal fluctuations with the characteristic kinetic energy of *k_B_T* (where *k_B_* is the Boltzmann’s constant and *T* is the absolute temperature) play a significant role. The self-assembly of viruses, including protein folding and the formation of a protein capsid and lipid bilayer membrane, is controlled by hydrophobic forces (i.e., the repulsing forces between hydrophobic particles and regions of molecules) in a water environment. Hydrophobic forces are entropic, and they are driven by a system’s tendency to attain the maximum disordered state. On the other hand, in information systems, entropic forces are responsible for erasing information, if the energy barrier between two states of a switch is on the order of *k_B_T*, which is referred to as Landauer’s principle. We treated hydrophobic interactions responsible for the self-assembly of viruses as an information-processing mechanism. We further showed a similarity of these submicron-scale processes with the self-assembly in colloidal crystals, droplet clusters, and liquid marbles.

## 1. Introduction

The recent COVID-19 pandemic has focused the attention of biophysicists and of the broad research community on viruses. Indeed, viruses constitute an amazing class of biological objects due to their intermediate position between living and non-living objects. Viruses have a genome encoded in a nucleic acid (either RNA or DNA) and replicate themselves; however, they do not possess the complex biochemical molecular apparatus needed for the translation and transcription of the genetic information. Instead, they use the apparatus of an invaded living cell. In addition, viruses do not have a metabolism or maintain homeostasis, which disqualifies them from being considered true living objects. On the other hand, parts of viruses have common features with physico-chemical self-assembling supramolecular structures, such as micelles, liposomes, and vesicles. This makes a thermodynamic analysis of the virus life cycle a significant objective for gaining general insights on self-assembly processes in physical chemistry.

While the length of time that virus particles can survive in dry conditions remains debated, they clearly prefer a water environment [1,2]. The recent study by van Doremalen et al. [3] showed that the SARS-CoV-2 virus remained stable in water droplets suspended in air for an extended time. Earlier studies have suggested that viruses containing lipids are more viable in moist air than in dry air [4]. The latter seems clear because the viruses can serve as nuclei of condensation of water vapor.

Understanding the effect of temperature and air humidity on the survival of airborne viruses is very important for preventing the spread of infectious diseases. Harper [5] found that influenza and several other viruses survived better at low temperatures and high relative humidity (RH). Similar laboratory observations were made by other researchers [6,7,8,9,10]. Viruses can survive on human skin and on household and contaminated surfaces [10,11,12]; however, they do not survive on the surface of ordinary materials woven from cotton fibers. All these observations suggest that viruses need water or moisture to survive [13].

Many viruses use atmospheric microdroplets to migrate for significant distances. Reche et al. [11] estimated the downward flux of viruses in aerosols created by sea spray on the order of 10^9^ per m^2^ per day. These viruses from marine sources were transported in the atmosphere for significant distances. Note that the number of viruses in seawater is gigantic. According to some estimates, every liter of seawater on Earth contains 10^11^ virus particles.

Most living organisms maintain the stable conditions needed for their survival through a complicated process referred to as homeostasis. Viruses do not maintain their own homeostasis. Our hypothesis is that viruses’ preference for an aquatic environment is related to their need to maintain thermal stability, in particular, one required for information processing during their “life cycle.”

**Figure 1:**
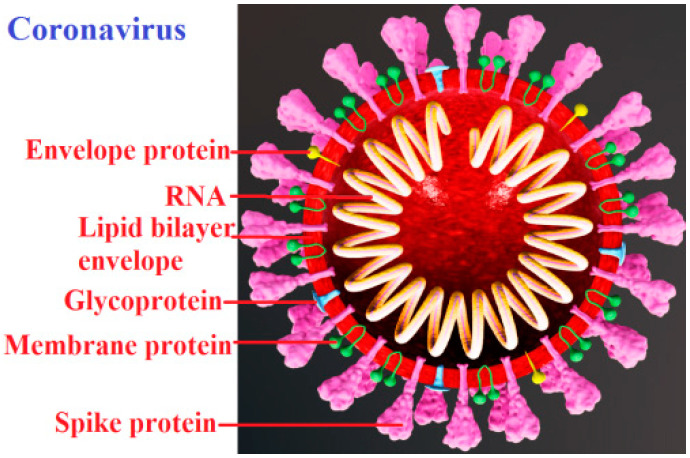
Schematic of an enveloped coronavirus (redrawn from https://www.scientificanimations.com/wiki-images).

Virus particles (virions) can be viewed as nanoparticles (Figure 1); however, viruses are built of biopolymers (nucleic acids and proteins) and other organic components such as lipids. These organic compounds are sensitive to environmental conditions. Many viruses, such as coronaviruses, are inactivated at temperatures of 55–60 °C [10,12]. From a thermodynamic point of view, virions are mesoscale structures. With a typical linear size between 20 nm and 300 nm, they are much larger than a typical atomic scale, but they are still much smaller than macroscale structures because their molecular masses (usually ranging between 10^6^ Da to 10^9^ Da) are much smaller than the Avogadro number (*N_A_* = 6.08 × 10^22^). As far as their genetic information, the genome of one of the smallest porcine circovirus (17 nm) is built of 1760 base pairs, whereas that of the largest Pandora virus (micron-sized) is built of 2.8 × 10^6^ base pairs.

The length scale between 1 nm and 0.1 mm, which is larger than an atomic scale (1 nm or less) but still smaller than a macroscale is often called the mesoscale. Mesoscale objects include systems that are of submicron size in at least one dimension, such as nanoparticles and structural elements of some soft condensed materials such as foams and gels. Another class of objects, which are sometimes classified as mesoscopic, includes systems in a near-critical state that have mesoscopic correlation lengths of spontaneous fluctuations. This is because a near-critical system may have a very large fluctuation length, so the effect of thermal fluctuations on them is comparable with that in much smaller regular systems.

A characteristic feature of mesoscale systems is that spontaneous fluctuations from equilibrium can play a significant role in them. Thus, for a small mesoscale oscillator in thermal equilibrium with the environment $\ddot{x}+\omega^{2}x=0$, whose position is characterized by the coordinate *x*, collisions with molecules will result in the mean square position being equal to $\langle x^{2}\rangle =k_{B}T/\left(m\omega^{2}\right)$ due to random thermal fluctuations.

## 2. Viruses as Thermodynamic and Information Processing System

Here we will discuss various stages of the life cycle of a virus and involved information-processing considerations.

### 2.1. Protein Folding

Protein folding is mostly controlled by hydrophobic forces, since hydrophobic elements of amino acids tend to pack into the protein’s interior. All 20 amino acids that form proteins can be arranged by their hydrophobicity according to hydrophobicity scales, although the methods of arrangement can vary. Thus, molecular dynamics (MD) simulations suggest that eight amino acids (Ile, Ala, Phe, Leu, Met, Pro, Val, and Trp) are hydrophobic, with water-contact angles up to 121.8°, four (Cys, Gly, Thr, and Ser) are hydrophilic, and eight (Tyr, His, Lys, Gln, Asn, Glu, Arg, and Asp) are superhydrophilic, and provide complete wetting [14].

It has been demonstrated that protein folding is often dominated by self-organized criticality (SOC), leading to fractal dependencies of the folded protein’s radius of gyration on its length (the number of amino acids) [15]. SOC is a particular type of self-organization found in many systems that leads to a near-critical behavior. It has characteristic quantitative signatures such as the one-over-frequency noise and fractal properties. In the case of polymer molecules, the dependency of the radius of gyration, *R*, on the length of the macromolecule, *N*, (or on its mass or volume) is considered, $R\propto N^{a}$. For a linear (unfolded) molecule, *a* = 1, while for a perfectly folded one, *a* = 1/3. However, polymer molecules are usually bent, and the dependency of *R* on *N* is non-linear. The Flory theory of a random polymer chain with excluded volume predicts the value of the power exponent of *a* = 3/5 (or *a* = 0.588 based on more elaborated renormalization arguments) [16].

For a protein consisting of only hydrophilic soluble amino acids, one could expect the same power exponent of *a* ≈ 3/5. At the opposite end, for perfectly hydrophobic insoluble amino acids, the power exponent of the linear scale dependency is *a* = 1/3. In practice, most proteins are built of both types of amino acids, and the power exponent of *a* = 2/5 has been reported experimentally [17,18]. The fractional dimension indicates that a fractal structure may characterize native-state (folded) polymers, and it serves as evidence of the SOC mechanism driven by hydrophobic forces [19].

As far as the information content of the proteins, this is a controversial topic. According to the widely accepted “Anfinsen’s dogma,” the native (folded) structure of a protein is completely determined by its amino-acid sequence [20]. This would imply that the amount of information describing the ternary (native) structure of a folded protein of *N* amino acids is not different from the amount of information in the genetic code, which encodes its linear (or primary) structure. The latter can be estimated as:

(1) $$H_{gen}=\log_{2}\left(20^{N}\right)=4.322N$$

given that there are 20 amino acids. However, the number of all possible native configurations of a protein is much larger. Thus, three stable conformation angles in each of two bond angles (φ and ψ) per peptide bond would imply 3^2N^ variants, so the fast folding kinetics constitute the so-called Levinthal paradox [21]. On the basis of these numbers, the information content of a folded protein molecule is estimated as:

(2) $$H_{native}=\log_{2}\left(3^{2N}20^{N}\right)=7.492N$$

The difference is the information produced and erased during folding due to the effect of the hydrophobic forces is:

(3) $$\Delta H_{folding}=H_{native}-H_{gen}=2N\log_{2}\left(3\right)=3.17N$$

### 2.2. Self-Assembly of Proteins and Lipids into Supramolecular Structures

Besides the information in the proteins themselves, additional information describing viruses can be found in the structure of their capsid and in the arrangement of proteins and other parts of viruses. In addition to protein folding, hydrophobic forces drive other self-assembly processes in viruses. Thus, the assembly of the capsid (a shell) is controlled mostly by hydrophobic interactions, while directional specificity is imposed by electrostatic, van der Waals, and hydrogen-bonding forces.

The capsid can often have an amazingly symmetric form, such as spherical or icosahedral, with the number of proteins proportional to 60. Various thermodynamic and kinetic models of self-assembly have been suggested, including those based on the Becker–Döring rate equation [22] and the low of mass action.

Note that the information entropy, symmetry, and algorithmic complexity are three related but different concepts. While information entropy is calculated as a binary logarithm of the number of possible combinations, complexity is a more elusive concept. A random set corresponds to maximum possible information, which is required for its description. However, in practice, only “useful” information is often important, and is associated with the complexity. In this sense, a random set is least informative. The so-called Kolmogorov complexity (KC) is defined as the length of the shortest algorithm needed to describe a system; however, although intuitive, the KC is incomputable, which limits its usefulness [23]. Consequently, those factors which erase information (such as thermal fluctuations and entropic forces) can simultaneously lead to symmetry and increased algorithmic complexity.

Many viruses are enveloped into a lipid bilayer membrane. The membrane consists of amphiphilic lipid molecules with their hydrophilic heads directed outside toward the water environment and their hydrophobic tails directed inward toward each other, forming a dual-layer shell. The process is characterized by a critical concentration of self-assembly (also referred to as the critical micelle concentration). The lipid bilayer self-assembly is also driven by hydrophobic forces [24].

For simplicity, we will consider the entropy associated with the capsid or lipid membrane self-assembly as that of phase separation. The latter can be calculated as the entropy of mixing:

(4) $$\Delta H_{SA}=-\overset{N}{\underset{n=1}{\sum }}X_{n}\ln X_{n}$$

where *X_n_* is the concentration of a phase number *n*.

### 2.3. Bio-Specific Interactions

There is another important informational aspect of virus protein self-assembly. The reproduction of a virus depends on its ability to enter an appropriate living cell and use its molecular machinery for copying itself. The ability of a virus to invade a cell is controlled by bio-specific interactions between virus membrane proteins and protein receptors at the membrane of the cell. Bio-specific or ligand-receptor (LR) forces are short-range (<1 nm) interactions between proteins dependent on their spatial structure and resulting from a combination of local ionic, H-bonding, and hydrophobic interactions between the amino-acid groups or subgroups [24]. An example would be the interaction between an antigen and an antibody generated by the immune response of an invaded organism.

The LR interaction is often described as a “key–lock” type of interaction. It is extremely difficult to predict whether two particular proteins would have a bio-specific interaction between them, so that one serves as a “key” corresponding to another’s “lock.” However, the ability of virus proteins to interact with cell receptors is what drives viruses’ evolution. Whether a virus protein corresponds to a cell receptor is the only “useful” information for this purpose, as opposed to the information about the protein’s structure.

Generally speaking, one can present an amino acid $a_{n}$ as an element of a set of amino acids $a_{n}\in A$, where 0 < *n* ≤ 20. The total number of theoretically possible proteins of length *k* is 20^k^. All lengths 1 ≤ *k* ≤ *N* should be considered. After that, a protein build of *N* or fewer amino acids can be presented as an element in the space of proteins $P\equiv \prod_{k=1}^{N}A^{k}$, which is a Cartesian product of amino-acid sets. The LR interactions between two proteins $p_{q},p_{r}\in P$ can be represented by a commutation matrix B≡P×P, which may be defined in such a way that $B\left(p_{q},p_{r}\right)=1$ if the interaction exists, and $B\left(p_{q},p_{r}\right)=0$ otherwise. The amount of information in matrix B is then equal to the number of elements in the square matrix with $P=\prod_{k=1}^{N}20^{k}$ rows and columns:

(5) $$H_{LR}^{*}={\left(\prod_{k=1}^{N}20^{k}\right)}^{2}.$$

Of course, such a matrix **B** should have an enormously huge size; however, only a small fraction of matrix elements would be different from zero, because each protein interacts only with a relatively small number of other proteins.

If the total nomenclature of cell receptors contains *M* proteins, and the virus includes *p* proteins, then the informational describing the LR interactions of the virus is estimated as a much smaller number:

(6) $$H_{LR}=p\log_{2}\left(M\right)$$

Moreover, from the viewpoint of virus reproduction and evolution, the information about membrane protein interaction with cell receptors is much more significant than the primary amino-acid structure of the protein, containing the information $H_{gen}$. Computational methods that retrieve correlations in large datasets have been recently developed [25]. They include the topological data analysis, which is used to reduce the dimension of high-dimensional datasets using persistent homology and to find low-dimensional structures with reproducible topology (e.g., the Betti numbers describing the rank of homology classes).

Thus, in all considered cases, we encounter similar situations. During the transition to a new stage in the virus life cycle (transcription/translation, self-assembly of protein and lipid structures, invasion of a host cell), the configurational space of the virus expands significantly, therefore, its description should require a much larger amount of information. However due to the action of external forces (in most cases these are the hydrophobic forces), the insignificant information is effectively erased.

### 2.4. The Information Life Cycle of a Virus

We can now present the life cycle of the virus as an information process, at every stage of which information is processed and erased. It has already been suggested that energy and information play a role in the life cycle of living organisms [26]. Kycia related the Landauer principle, which he treats as a special case of a more general Galois connection, to the DNA and RNA processing in vivo and in vitro [27].

First, the nucleic acid of a virus is reproduced inside an invaded cell involving the processes of the transcription and translation. This stage is dominated by the genetic code information of $H_{gen}=4.322N$ bits. Second, the proteins are produced and form their native configuration structure. While the complete description of the native configuration would require $H_{native}=7.492N$ bits of information, this information is contracted by $\Delta H_{folding}=3.17N$ bits. This is because protein folding is driven by forces, mostly hydrophobic, that reduce the primary (unfolded) structure into a unique native (tertiary or folded) structure. The folding–unfolding transition is usually reversible. The unfolding (denaturation) can be triggered by heating or by introducing an aggressive chemical environment (e.g., household detergent or alcohol are common denaturants), which would break hydrophobic bonds between amino acids, but would preserve the much stronger peptide covalent bonds forming the primary structure of the protein.

Third, the shell and other elements of the virus particle are formed. This often results in geometric shapes characterized by a high degree of symmetry. In many instances, the self-assembly is similar to the phase separation, e.g., insoluble molecules aggregate together when their concentration exceeds a certain critical level. This process can be roughly characterized by the information entropy of mixing, $\Delta H_{SA}=-\sum_{n=1}^{N}X_{n}\ln X_{n}$. Again, the process is driven largely by hydrophobic forces.

Fourth, the virus should invade a living cell using the cell’s receptor proteins, which would grant its entrance into the cell. Such receptor proteins should fit the membrane protein of the virus, causing a bio-specific reaction. At this stage, the information content reduction is characterized by $\Delta H_{LR}=H_{LR}^{*}-H_{LR}={\left(\prod_{k=1}^{N}20^{k}\right)}^{2}-p\log_{2}\left(M\right)$.

**Figure 2:**
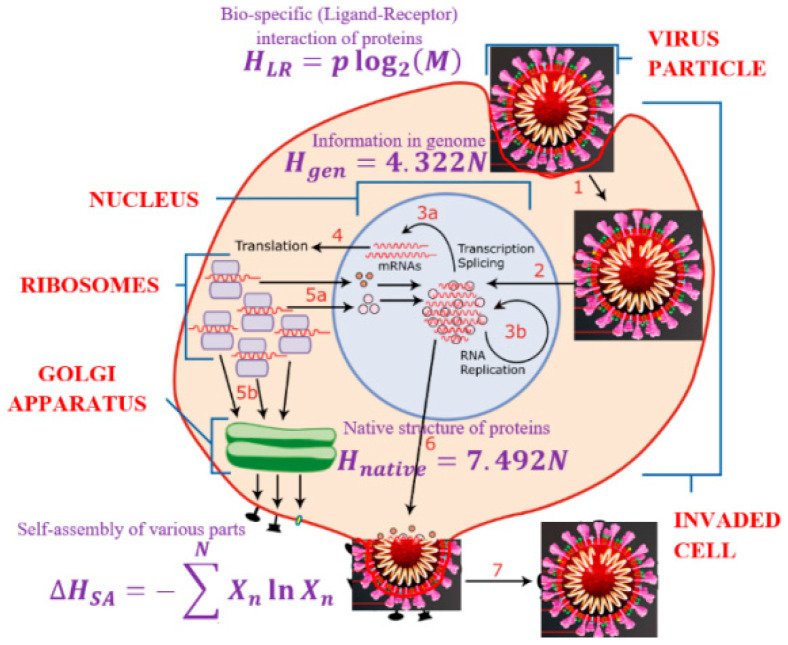
Schematic of the life cycle of a virus: (1) invasion of a cell, (2) transcription, (3) replication, (4) translation, (5) protein self-assembly, (6) exiting cell, (7) virion outside the cell (parts of the image redrawn from https://www.scientificanimations.com/wiki-images).

At every stage of the life cycle of the virus (Figure 2), the configurational space of the virus expands significantly, requiring a much larger amount of information for the description of the virus’ state. However, due to the action of an external force, the insignificant information is effectively erased, leading to a much smaller amount of significant information. In many cases, these external forces are hydrophobic forces. Hydrophobic forces are usually described as entropic forces that drive a system to the state of maximum disorder or maximum entropy by applying random thermal fluctuations [24].

The common understanding of the entropic molecular origin of hydrophobic forces is that polar water molecules tend to form dynamic tetrahedral networks due to H-bonds. An introduction of insoluble non-polar groups results in the disruption of the network, with water molecules attempting, due to random thermal fluctuations, to restore the tetrahedral network by isolating the hydrophobic or non-polar molecules, effectively leading to their mutual attraction, which is manifested as a hydrophobic attraction force. Entropic interactions are dependent on the thermal motion of molecules, whose effect is therefore proportional to the local absolute temperature.

Unfortunately, it is very difficult to directly measure the information content at different stages of the virus life cycle, since there are no methods of such measurement currently available. Indirect judgment on the role of entropic forces can be obtained from the temperature dependencies of reaction rates. This is because the entropic contribution to the changing Gibbs energy during a chemical reaction, ΔG=ΔH−TΔS, is proportional to the temperature, TΔS, as opposed to the non-entropic (enthalpic) term ΔH.

### 2.5. Thermal Considerations and Landauer’s Principle

Given that at every stage of its life cycle, a virus’ transformation involves the erasing of information, so the thermal cost of erasing information should be taken into account. Irreversibly erasing one bit of information at the ambient temperature of *T* involves energy dissipation, the lower limit of which is bounded by the amount of $\Delta E=k_{B}T\ln2$. This is known as Landauer’s principle [28]. In order to store one bit of information, a physical storage mechanism should exist that forms a switch with two stable states that correspond to 0 and 1. At a given temperature, the two states should be separated by an energy barrier with a height of ΔE or higher. Otherwise, thermal fluctuations with the energy on the order of $k_{B}T$ would change the position of the switch and the information will be lost.

Note that while the standard formulation of Landauer’s principle involves a binary calculation (and Boolean logic), the genetic code of the DNA or RNA consists of four bases. Many-valued logic is used in those areas of physics where no clear binary dichotomy exists [29]. A generalization of Landauer’s principle for a many-valued logical computation has been suggested [30]. Besides that, generalizations of the Landauer principle as a special case of a more general Galois connection may also be relevant to DNA/RNA computation [27].

On the other hand, storing information involves erasing the previous state of the storage. When one bit of information is erased, the switch should be transferred into a different state. For that end, an energy exceeding the barrier ΔE must be applied. This energy is irreversibly dissipated, because the switch should rest in its new position. From these considerations, we conclude that the energy cost of storing the information is proportional to the ambient temperature. One can therefore expect that natural systems that rely heavily on information processing, such as viruses, would have the tendency to operate at lower temperatures.

While the intensity of entropic forces is expected to grow with increasing temperature, elevated temperatures are not beneficial for viruses. The thermal fluctuations that erase the insufficient information can also erase the needed information. Elevated temperatures may lead to protein denaturation and other effects, destroying the self-assembled structures. The kinetics of thermal inactivation of a virus is characterized by the rate of decrease of the substrate population $k_{T},$ defined as:

(7) $$\frac{dA}{dt}=-k_{T}A$$

where *A* is the virus population. The rate of thermal inactivation is governed by the Eyring equation:

(8) $$k_{T}=\frac{k_{B}T}{h}\exp\left(-\frac{\Delta G}{k_{B}N_{A}T}\right)=\frac{k_{B}T}{h}\exp\left(\frac{k_{B}N_{A}T}{\Delta H}\right)\exp\left(\frac{\Delta S}{k_{B}N_{A}}\right),$$

where *h* is the Planck constant, and ΔG=ΔH−TΔS is the change of the molar Gibbs energy of the population as it goes to the inactivated state, ΔH is the activation molar enthalpy, and ΔS is the activation molar entropy [27]. Therefore, the rate of inactivation of viruses increases dramatically with heating.

Besides heating, ultraviolet (UV) light irradiation leads to rapid virus inactivation [31]. Tseng and Li [32] reported that the so-called UV germicidal irradiation at wavelengths near 253.7 nm (close to the maximum absorption wavelength of a DNA molecule) destroys viruses. Airborne aerosol viruses are also deactivated by solar UV radiation [33] and by far-UVC light (in the wavelength range of 207–222 nm) [34]. The energy of the UV (wavelength λ≅200 nm) quantum is $E=\frac{hc}{\lambda }≅10^{-18}\;J.\;$According to Landauer’s principle, the energy necessary for erasing of one bit of information at the room temperature T≅300 K equals $E_{L}≅ln2k_{B}T≅1.3\times 10^{-21}J$. It is seen that the energy of a single quantum of UV light is sufficient for erasing ca. $10^{3}\;\mathrm{bits}\;\mathrm{of}\;\mathrm{information}$.

Viruses do not have their own mechanisms for homeostasis, and therefore are unable to maintain the optimum temperature for their operation. Instead, they rely on external mechanisms. The preference for an aquatic environment, which is characterized by much smaller thermal fluctuations than air at the same temperature, may be one such mechanism, with water serving as a thermal reservoir that stabilizes virus’ temperature.

Erwin Schrödinger [35] noted than one of the objectives of nurture and metabolism is to create low-entropy products that are digested and maintain the low entropy of a living organism. Viruses do not have their own metabolism, and they rely on hydrophobic self-assembly forces to maintain their organization and prevent disintegration.

## 3. Mesoscale and Microscale Structures Whose Self-Assembly Is Facilitated by Water

Besides viruses, there are a number of self-organizing systems for which water facilitates self-assembly mechanisms. Some of these objects are macroscale, and their similarity with mesoscale systems is remarkable. Droplets themselves can lead to self-assembling structures; thus, the shape of a small liquid droplet near the freezing point is defined by topological laws, and they can become icosahedral, which makes them similar to the self-assembled capsid [36].

**Figure 3:**
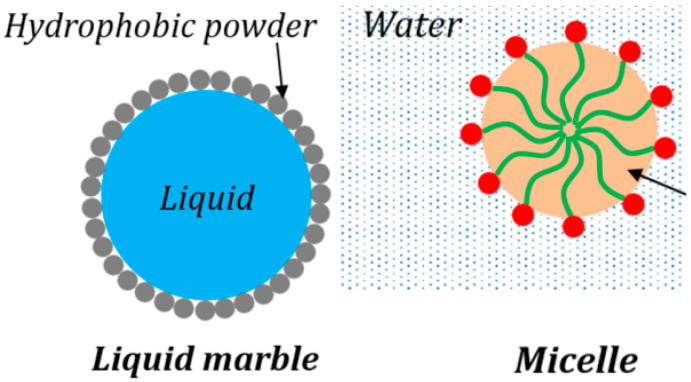
Liquid marble vs. micelle.

Most obvious is the geometric similarity between liquid marbles and micelles (Figure 3) [37]. Liquid marbles are formed by liquid droplets rolling upon a fine powder, so that the droplets are spontaneously coated with a layer of grains of the powder. It is energetically favorable for the particles to attach to the liquid–vapor interface. At small volumes, liquid marbles maintain an almost spherical shape, and they can be deformed, changed in size, manipulated, and transferred to different surfaces. In many aspects, it is easier to manipulate the marbles than droplets; in addition, the particle monolayer slows down liquid evaporation, while the liquid no longer wets any supporting surface [38]. A model macroscopic system with a composite liquid marble contacting with hydrophobic/hydrophilic particles imitating the entry of viruses into living cells was suggested by Roy et al. [39]. Composite marbles absorbed hydrophilic polymer particles, but prevented hydrophobic particles from entering their core. The swallowing of hydrophilic particles by composite marbles resembles the penetration of viruses into living cells.

Micelles are nanometer-scale structures formed in a colloidal system when surfactant molecules arrange themselves into a spherical structure. Surfactants usually have a hydrophilic or ionic head and a hydrophobic–aliphatic tail. As surfactants are added to a colloid, the surface energy of the interface between the phases continues to decrease. Once surfactants reach a critical concentration (called critical micelle concentration), they can spontaneously agglomerate so that their heads are all arranged toward one phase of the colloid, and their tails toward the other phase. When the size of the surfactant molecules are within a favorable limit, the agglomeration results in a spherical structure [40]. Powder-coating modifies the surface energy of liquid marbles, while surfactants do the same for micelles. The formation of liquid marbles and spherical micelles is driven by the minimization of surface energy.

**Figure 4:**
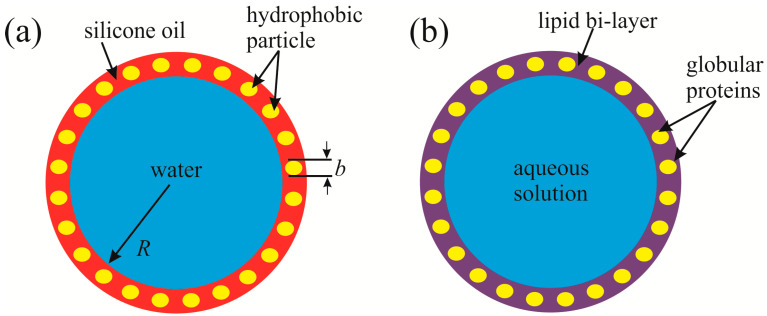
Sketch demonstrating the interfacial resemblance of a composite liquid marble (**a**) and living cells (**b**); the radius of the marble R≅2.5 mm; the primary diameter of the hydrophobic fumed fluorosilica particles b≅30 nm.

Bi-liquid, core-shell, or composite marbles, i.e., water marbles coated with a thin layer of oil comprising solid hydrophobic particles, were introduced recently [41,42,43]. The structure of the external interface of a composite liquid marble resembles that of living cells or virus membranes, built of lipid bilayers (including cholesterols) and containing intrusions of globular proteins and fragments of the cytoskeleton (Figure 4a,b). In both composite marbles and living cells, water is enveloped by a hydrophobic layer filled by heterogeneities.

The composite marbles swallow solid hydrophilic entities, resembling the process of viral shedding that occurs via so-called viral budding [44]. We do not exaggerate the physical similarity of the composite marbles and living cells; however, the core-shell marbles exemplify the budding of enveloped viruses within a macroscopic system. Thus, composite marbles may supply an interfacial model of a living cell.

**Figure 5:**
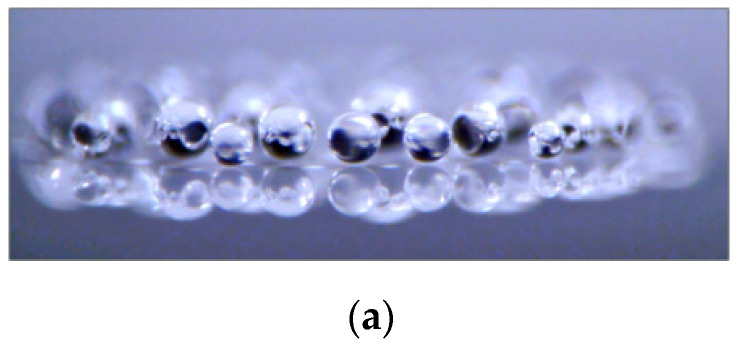

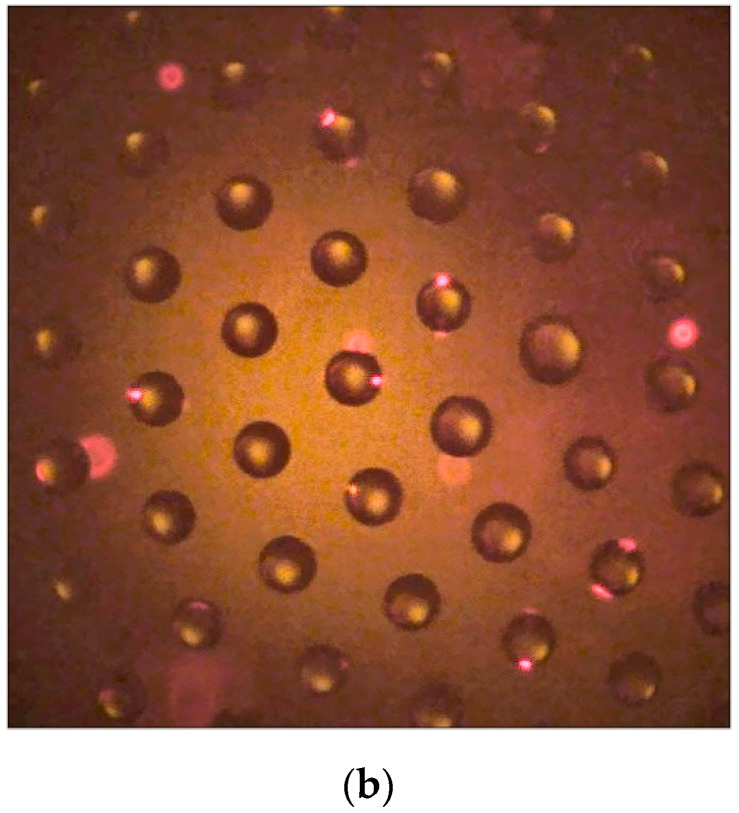
Typical images of a levitating droplet cluster: (**a**) side view, (**b**) top view of a cluster with incorporated *Chlorella vulgaris* cells.

Another amazing self-assembled water object is the droplet cluster, consisting of condensed droplets in the range of about 10–100 µm in size levitating over an ascending vapor-air flow and self-assembling into a regular hexagonally symmetric (honeycomb-like) structure [45,46]. Interactions that control the self-assembly of the droplet cluster are water phase transitions (condensation and evaporation) and aerodynamic forces from the flow of a mixture of water vapor and ambient air [47]. The water cluster can be used for in situ monitoring of airborn microdroplets containing small biological objects [48,49]. Thus, living cells are adsorbed at the liquid–gas phase interface due to hydrophobic forces, which are also crucial for the cell membrane (Figure 5).

## 4. Conclusions

From a physicist’s point of view, virus particles are just nanoparticles. However, unlike engineered inorganic or organic nanoparticles, a water environment is crucial for viruses. Hydrophobic forces facilitated by water are responsible for most self-assembly effects throughout the life cycle of the virus. These forces are entropic and they intensify with increasing temperatures. However, at elevated temperatures, viruses inactivate and are destroyed due to protein denaturation. Therefore, an optimum temperature is required. Since viruses do not maintain homeostasis, they rely on an external stabilizing by the thermal reservoir formed by an aquatic environment. Drying of microdroplets containg viruses is viewed as an important mechanism to limit the spreading of viral respiratory diseases [49,50].

The life cycle of a virus can be viewed as an information-processing cycle, with the information of nucleic acids being converted into the information about the native structure of proteins, then about self-assembled structures built of proteins and about bio-specific interactions of proteins, for example, with invaded cell receptors. The action of entropic forces, facilitated by thermal fluctuations, can be viewed as information processing—in most cases, the erasing of unneeded information while keeping the needed information. In view of Landauer’s principle, which relates the energy needed for irreversible information processing to thermal fluctuations, the role of entropic hydrophobic forces can be viewed as the facilitation of the information-processing cycle.

Besides its role in protein folding and the self-assembly of such structures as the protein capsid and lipid membrane, water is prominent in self-assembly of many other colloidal systems, including micelles, liposomes, liquid marbles, and droplet clusters.

## Data Availability

Data sharing not applicable.
